# A Simple Imaging Device for Fluorescence-Relevant Applications

**DOI:** 10.3390/mi9080418

**Published:** 2018-08-20

**Authors:** Shih-Jie Lo, Chen-Meng Kuan, Min-Wei Hung, Yun Fu, J. Andrew Yeh, Da-Jeng Yao, Chao-Min Cheng

**Affiliations:** 1 Institute of Nanoengineering and Microsystems, National Tsing Hua University, Hsinchu 30013, Taiwan; roxyjay0406@gmail.com (S.-J.L.); kcmeng30@gmail.com (C.-M.K.); jayeh@mx.nthu.edu.tw (J.A.Y.); 2 Instrument Technology Research Center, National Applied Research Laboratories, Hsinchu 30013, Taiwan; niiat690310@gmail.com; 3 Department of Dermatology, Chang Gung Memorial Hospital Linkou Medical Center, Taoyuan 33305, Taiwan; amyjoy791126@hotmail.com; 4 Institute of Biomedical Engineering, National Tsing Hua University, Hsinchu 30013, Taiwan

**Keywords:** cellphone, fluorescence imaging device, nucleotide analysis, sperm analysis

## Abstract

This article unveiled the development of an inexpensive, lightweight, easy-to-use, and portable fluorescence imaging device for paper-based analytical applications. We used commercial fluorescent dyes, as proof of concept, to verify the feasibility of our fluorescence imaging device for bioanalysis. This approach may provide an alternative method for nucleotide detection and semen analysis, using a miniaturized fluorescence reader that is more compact and portable than conventional analytical equipment.

## 1. Introduction

In the last decade, point-of-care (POC) diagnostics research has persistently pursued the World Health Organization (WHO) standards to fulfill analytical demands, for resource-constrained regions [1,2]. Among available POC technologies, paper based analytical devices (PADs) are considered one of the most promising solutions due to several advantageous properties, including inexpensiveness, ease of use, flexibility, high level of compatibility with current fabrication techniques, and easy disposal by incineration [3]. Successful demonstrations of PADs have involved the use of various methodologies, including colorimetric assay, immunoassay, fluorescent assay, chemiluminescence assay, surface-enhanced Raman spectroscopy, and electrochemical analysis [4,5,6,7,8,9,10]. In regards to medical diagnostics, the development of PADs has largely focused on quantifying small compounds related to the detection and quantification of metabolic disease-related compounds (e.g., glucose), antigens or enzymes (such as alanine transaminase (ALT), prostate-specific antigen (PSA), microorganisms (e.g., Mycobacterium tuberculosis), viruses (e.g., hepatitis C virus), nucleotide analysis, and even semen analysis [9,10,11,12,13,14,15,16,17,18,19,20,21,22]. 

With advances in microelectromechanical systems (MEMs), cellphones are now capable of sensing and processing quality images with remarkable processing speed [23]. Resulting images and data taken “in the field” may be analyzed on site or readily shared for additional inspection and review [24,25]. There were an estimated 6.9 billion existing cellphone subscriptions (2013), which were expected to grow to as many as 8.0 billion subscriptions by 2016, paving a path for the integration of cellphones into POC and personalized medicine [26]. Cellphone-based devices (CBDs), that are lightweight and compact, may serve many roles as readers for immunoassays and lateral flow tests, electrochemical and surface plasmon resonance-based bio-sensing, microscopy, cytometry, colorimetric detection, and other healthcare monitoring procedures [27,28,29,30,31,32,33,34]. At present, CBDs that have been successfully implemented in cell or microorganism inspections have relied on fluorescent assays and organic-based compounds, quantum dots, or nanoparticles. Only a few studies have implemented nucleotide analysis and semen evaluation via a cellphone-based fluorescent approach [28,35,36,37]. While CBDs can be relatively sophisticated from an engineering perspective, we believe that our elegant approach to their use, is something that more traditional life sciences researchers can connect to and expand upon. 

Here, we demonstrate a simple portable fluorescence imaging device that employs a common DNA dye and a viability dye for semen evaluation, as proof of concept. This system leverages the recording capacity of a commercial cellphone camera, in combination with a PAD, to accomplish detection and analysis of various concentrations of DNA and sperm. This device is lightweight (203.4 g) and inexpensive, compared to conventional fluorescence readers. It also uses a plastic shell to prevent interference from ambient light and provide a uniform light source for quantitative use, as well as a light filter to optimize the recorded wavelength, for more straightforward analysis. Moreover, our device allows users to select varied UV-LED and light filter combinations, according to their needs, which may provide distinct opportunities for additional forays into fluorescence-based detection (e.g., fluorescent protein level measurement). It has been our intent to provide a relatively simple and versatile CBD, which can be used without rigorous space or environmental demands and is capable of diverse implementation [29]. While we have made significant advances, we encountered some challenges described herein. This study is expected to benefit POC diagnostic fields, by demonstrating prototypical lightweight instrumentation for fluorescent quantification.

## 2. Materials and Methods 

### 2.1. Portable Fluorescent Image Recording Device

The device consisted of the following mechanical components: UV-LED, an LED-driving circuit board, a battery, a light adjuster, and a light filter. The dimensions of the prototype were 15.5 × 9.5 × 5.2 cm^3^, which provided flexibility for changing optical components. The UV-LED (SSL-LXTO46UV2C, LUMEX, Carol Stream, IL, USA) had a central wavelength of 405 nm and a power of 100 mW. The circuit board was designed to tune light intensity through pulse-width modulation (PWM), i.e., resistance applied determined light magnitude.

### 2.2. Chemicals and Materials

The paper-based analytical device was produced using Whatman qualitative paper (Whatman grade No. 1 filter paper No.:1001-185, Pittsburgh, PA, USA) and was fabricated via a wax printing method with a wax printer (Phaser 8560DN, Xerox, Norwalk, CT, USA). Calcein AM dye was purchased from Thermo Fisher Scientific Inc. (No. C1430, Taipei, Taiwan). U-safe fluorescent probe was purchased from Bio-Genesis Technologies (No. DBU-009, Taipei, Taiwan) and 1 Kb DNA ladder was purchased from Protech Technology (M1-1KB, Taipei, Taiwan). Cameras within a Sony Ericsson K610i (2 megapixel; Sony Mobile Communications), an HTC Butterfly (8 megapixel; HTC Corporation), an iPhone 5, and a 5S (8 megapixel; Apple Inc.) were used to capture the experimental images. The regulator (No.: LM78L05) on the LED-driving circuit board was purchased from Unisonic Technologies Co., Ltd. (UTC, Taipei, Taiwan). The N-type MOSFET (No.: P1004BD) was purchased from Niko Semiconductor Co., Ltd. (Niko-Sem, Taipei, Taiwan). The microcontroller unit (MCU; No.: PIC16F722) was purchased from Microchip Technology Inc. (Chandler, AZ, USA). Wavelength confirmation in the experiments was achieved using a miniature spectrometer (SD1200, OTO Photonics Inc., Taipei, Taiwan).

### 2.3. DNA-Stainging Assay and Sperm Analysis

DNA ladders were serially diluted with ddH_2_O to concentrations of 400, 200, 100, and 50 μg/mL, sequentially. The fluorescent probe (U-safe) was diluted 10-fold with ddH_2_O. Next, the diluted DNA ladders and the diluted fluorescent probe were mixed together at a 1:1 volume ratio. The final DNA concentrations of each mixture were 200, 100, 50, and 25 μg/mL. The final concentration of the fluorescent probe was diluted 20-fold. After 15 min of mixing time, 2 μL of the mixture was placed into each paper-based test zone. 

In our semen assay, Calcein AM dye was first diluted 50-fold with our dilution buffer. The dilution buffer was constituted with 10 mmol/L HEPES (4-(2-hydroxyethyl)-1-piperazineethanesulfonic acid), 150 mmol/L NaCl, and 10% BSA (Bovine Serum Albumin), with a pH value of 7.4. The diluted Calcein AM dye was then mixed with semen at a 1:1 volume ratio. After 30 min of mixing time, 6 μL of the semen mixture was placed into the test zone.

After receiving test mixtures, the paper device was allowed to dry naturally (approximately 20 min), and ddH_2_O was used to wash each test zone three times (i.e., 8 µL each time) in each assay. After the washing step, the test zones were imaged using our recording device.

### 2.4. Image Capture and Intensity Analysis

All experimental images were captured using the built-in camera software, in an array of different smartphones. Camera focus during image capture was set on the center of each single spot (bright area) for single-spot detection, and on the center of each four-spot test (dark area) for multi-spot detection, to diminish the intensity variations caused by the native auto-adjusting functions of each smartphone. Captured images were exported to a computer and saved in JPEG format. The mean intensity of each exported image was analyzed with free public software (ImageJ, Ver. 1.49q), and was presented as a mean value with a standard deviation (Mean ± S.D.). The Student’s *t*-test was employed to analyze the significant difference between two experimental groups. A *p*-value is the probability that the results from the sample data occurred by chance. In general, lower *p*-values indicate higher significant difference between two groups. A *p*-value of 0.05 indicates only a 5% difference, which is an acceptable difference, indicating that the data is valid.

## 3. Results and Discussion

### 3.1. Functionality of Fluorescence Imaging Device

**Figure 1:**
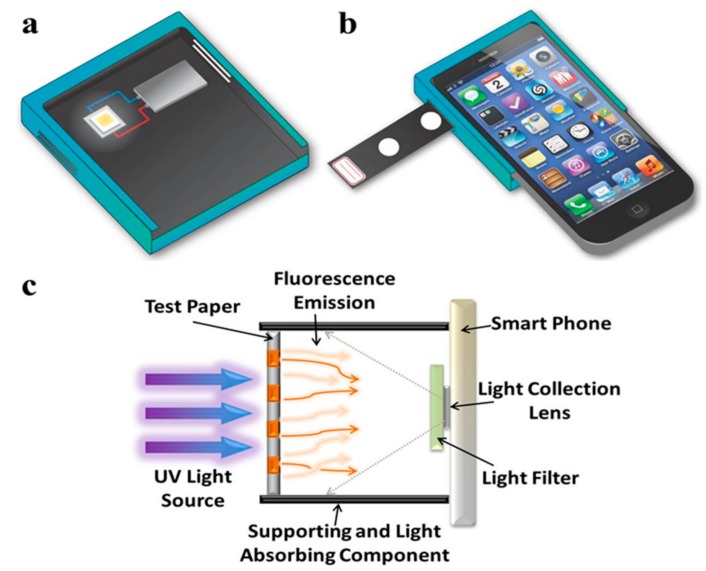
(**a**) A schematic of the desired portable image-recording device, which contains an LED (yellow square in the center of white square), a battery (rectangular with gradient gray), and a plastic shell with two slots on both sides. (**b**) A schematic of the desired portable device combined with a cellphone and a paper stripe. (**c**) A schematic of the basic design components for fluorescence detection, using the camera system of a cellphone.

**Figure 2:**
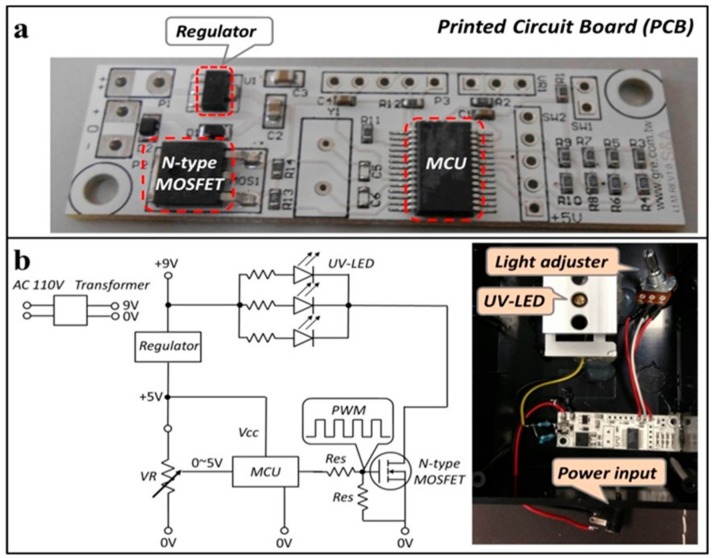
(**a**) The designed electronic components on a printed circuit board (PCB) and (**b**) the schematic diagram of the portable image recording device. The power input is linked with an AC 110 V transformer, and the light adjuster is operated to alternate the variable resistance (VR).

**Figure 3:**
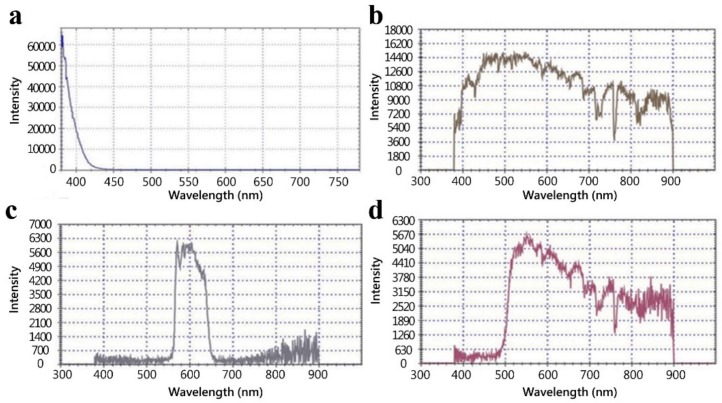
Wavelength measurement of the UV-LED and light filters. (**a**) UV-LEDs emit light with a peak intensity below 400 nm. (**b**) Sunlight was used as a light source with full wavelength. (**c** and **d**) The wavelength of sunlight penetrating through a band-pass 600 nm filter and a long-pass 500 nm filter, sequentially.

Figure 1a,b provide an overview of the portable imaging device, as follows: (i) UV-LED; (ii) a battery; (iii) a plastic shell with two slots on both sides for inserting a light filter; (iv) an observation hole for a paper device; and (v) a cellphone attachment. UV light (405 nm) from the LED chip penetrated through the PAD and light filter, and the final transmission light was recorded via a cellphone camera (Figure 1c). Additional elements included assembled electronic components on a printed circuit board (PCB) with a light adjuster and UV-LEDs in a black plastic box, for our fluorescence imaging device. The circuit on the PCB controlled UV-LED power output and manipulated light intensity (Figure 2). The regulator allowed us to modulate direct current (DC) voltage in the range of 9 V to 5 V, to accommodate a microcontroller unit (MCU). The function of the N-type MOSFET was to amplify power. Following pulse-width modulation methodology, the voltage signal was manually controlled by adjusting resistance, and could be converted to 8-bit digital signals corresponding to 0–100% of the duty cycle. The purpose of the power-modulated circuit was to regulate UV-LED intensity, and to determine the appropriate wavelength for excitation of fluorescent components. Peak wavelength from the manufacturer-provided UV-LED data sheet reported that the LED light emitted light ranging from 400 nm to 410 nm, but our measurements with a spectrometer indicated that the wavelength of emitted light had a peak intensity slightly less than 400 nm (Figure 3). We also employed sunlight as a standard reference to ensure normal spectrometer function; the wavelength of sunlight was measured, as shown in Figure 3b. In addition to confirming the emitted UV-LED wavelength, the wavelength of light penetrating our filters was also verified. Two light filters, band-pass 600 nm (600 ± 80 nm) and long-pass 500 nm (>500 nm), were selected for fluorescent light filtration in the DNA quantitative experiment and semen assay, sequentially. Figure 3c,d, depict the wavelength measurement of sunlight penetrating through band-pass 600 nm and long-pass 500 nm, sequentially. These measured results indicate that the filtered light wavelengths corresponded to wavelengths provided by the manufacturer-provided filter datasheets.

### 3.2. Fluorescence Detection Device for Nucleotide Analysis and Semen Analysis

**Figure 4:**
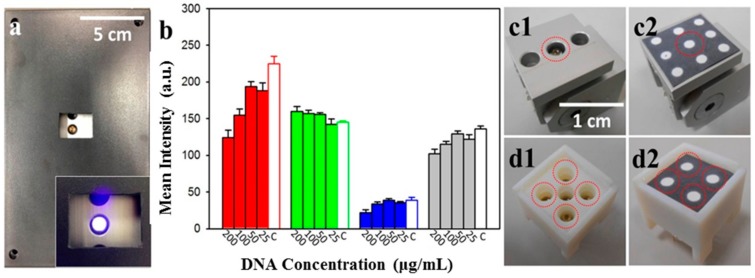
(**a**) The full top view of our device in operation. The hole of the lip is for image-taking with a cellphone lens, and the small insert image represents the light when switched on. (**b**) The intensity analysis of each RGB (i.e., Red, Green and Blue) channel and greyscale for different nucleic acid concentrations. The red channel intensity significantly increased when DNA concentration decreased (N = 8; mean ± standard deviation). The letter C means control group where the concentration is 0 μg/mL. (c and d) Two types of UV-LED modules were designed for single-spot (**c1** and **c2**) and multi-spot detection (**d1** and **d2**). The dashed circles mark the UV-LED site.

Images of PAD results were recorded through an observation hole (Figure 4a). We previously assured the viability of nucleotide analysis with PADs, by monitoring the fluorescence signals of nucleotides labelled with different fluorescent probes (non-specific fluorescent signals could be eliminated using a multiple washing approach), as described in Reference [7]. Various DNA concentrations mixed with U-safe fluorescent dye were placed onto our paper-based device. These samples were excited using the LEDs of our portable device, and fluorescence was recorded using different cellphone camera systems. ImageJ software was used to analyze the fluorescence intensity by splitting signals into red, green, and blue channels, as well as into simple greyscale (Figure 4b). Results from green channels, blue channels, and greyscale showed no scientific correlation with DNA ladder changes; however, increasing red channel signal intensity was correlated with a decline in DNA ladder concentration, so the red channel was chosen for the subsequent system test. The red signal change was attributed to the fact that the emission spectrum of U-Safe nucleic acid gel staining dye was located at the wavelength of orange light (597–620 nm) [38]. We noticed a negative trend of fluorescence, corresponding to the DNA concentrations. This may be due to the different binding mechanisms of the staining dyes used, and in particular their affinity for nucleic acids and substrate. We used these dyes for nucleic acids in our previous study [7]. The fluorescent probe that we used carried positive charges, to detect nucleic acids. In this experiment, we first mixed U-safe and DNA ladders together, before placing the mixture onto our paper substrate. The surface of our paper substrate expressed negative charges, contributed by hydroxy groups. Hence, we speculated on the presence of a competitive binding process between the fluorescent probe, the nucleic acid, and the paper substrate. In addition to single-spot detection, we also demonstrated multi-spot detection design to increase system utility, as shown in Figure 4c,d.

**Figure 5:**
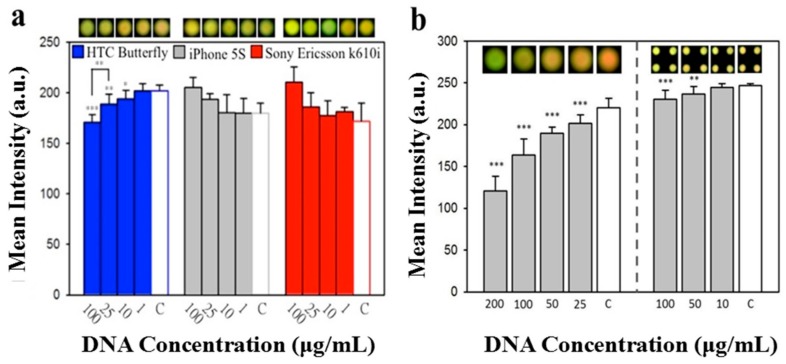
Intensity analysis from the images recorded using different cellphones. (**a**) Different cellphones were used to capture images; mean intensity corresponded to DNA concentration, but varied slightly between camera systems used. (**b**) Images recorded with an iPhone 5, showed the best sensitivity in our designed approach. The intensity demonstrated significant difference, compared with the control group of the device equipped with a single UV-LED (left part of chart). Although intensity of the device equipped with multiple UV-LEDs also expressed significant differences above 50 μg/mL of DNA concentration (right part of chart), the multi-spot recording should be modified for better performance. * *p* < 0.05; ** *p* < 0.01; *** *p* < 0.001 (Student’s *t*-test), indicating statistically significant differences compared with the control group (N = 8; mean ± standard deviation).

Different cellphone camera systems were tested for fluorescence analysis. Figure 5 indicates the results using an HTC Butterfly, iPhone 5S, Sony Ericsson k610i, and an iPhone 5 to record different levels of DNA ladder. Neither the Sony Ericsson k610i nor the iPhone 5S, demonstrated statistically significant differences between experimental groups and respective control groups. The HTC Butterfly and the iPhone 5, however, provided encouraging results with significant differences in signal intensities of DNA ladder, at concentrations of 10, 25, and 100 μg/mL, compared to their control group values (Figure 5a,b). The discrepancies between phones may have been associated with unique image-processing algorithms installed in each cellphone. In addition to discovering that some phones were better suited to this process, we discovered that a device with one UV-LED produced a better detection consequence, than a device with multiple UV-LEDs (Figure 5b), which may be partly due to automatic image-processing algorithms influences. Furthermore, the ratio of occupied test areas in the recorded image may contribute to this phenomenon, in that the analyzed results from our single UV-LED module were better than the results from our multiple UV-LED module. In other words, the total number of pixels relating fluorescent intensity via single UV-LED methodology, was greater than that via multiple UV-LED methodology. This meant that the dynamic range in the single UV-LED module was better than in that in the multiple UV-LED module. Nonetheless, we believe that this issue can be eliminated through fine engineering improvement, and is not an obstacle to future development.

**Figure 6:**
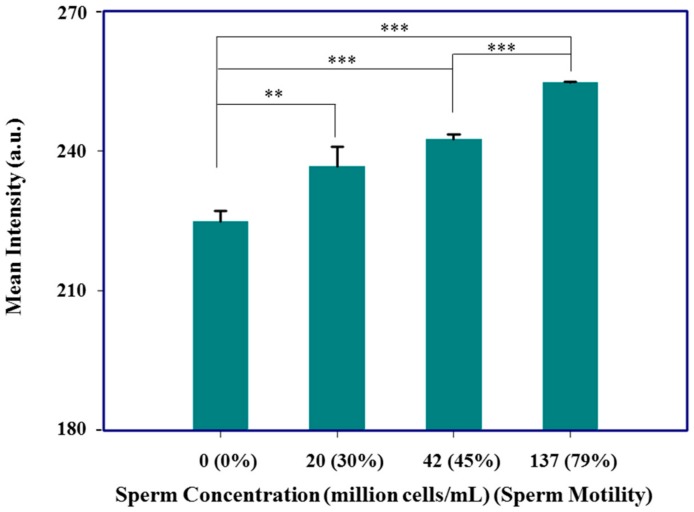
Fluorescence detection device for sperm analysis. Sperm analysis results revealed a relationship between serial sperm dilutions and motility, 0 (0%), 20 (30%), 42 (45%), and 137 (79%) million cells/mL (motility). ** *p* < 0.01; *** *p* < 0.001 (Student’s *t*-test), indicating statistically significant differences compared with the control group (N = 3; mean ± standard deviation).

In addition to nucleotide detection, this system also attempted to serve as a tool to evaluate sperm concentration and mobility (Figure 6). The infertility rate in developed countries is in the range of 10–15%, whereas the infertility rate in sub-Saharan African countries is in the range of 20–46% [18,38]. Infertility in men is associated with the total/mobile number, motility, and morphology of sperm, and microscopic inspection is the standard methodology to assess sperm quality in clinical centers [39]. However, this examination approach is not suited for the development of home-based sperm analysis, a process that typically requires professional training. For our device, we selected an iPhone 5 to record Calcein AM dye signal intensities (a common fluorescent viability dye), corresponding to different sperm concentrations and motility. We diluted our original boar sperm sample (136 million cells/mL), with serum dilution buffer to prepare sperm concentrations of 20 and 42 million cells/mL. Each group showed statistically significant differences in green channel signal intensities, in comparison to the control group.

## 4. Conclusions

In this paper, we successfully demonstrated the development of a fluorescence imaging device integrated with commercial cellphone technology and a paper-based analytical tool, for nucleotide detection and sperm analysis. Our device employed UV-LED light for fluorescent signal excitation, a power-modulated circuit board for modulating light intensity, and a light filter for optimizing fluorescent signal recording. We noted that the images recorded by different cellphone camera systems exhibited various results for analyzed fluorescence intensity, and that some phones were better suited to this approach. Moreover, while a device equipped with multiple UV-LEDs was developed to improve image recording efficiency, we found that a slight modification to this approach was required to improve fluorescence image analysis. We feel certain that variations between cellphone camera systems could be accommodated, by designing a cellphone application to manage image capture variations. Finally, although this platform may currently offer little toward resolving existing complicated diagnostic medical challenges, we believe it provides traditional biologists with an understanding of a new and highly versatile integrative tool, CBDs; that they might not otherwise be exposed to, and that might launch further and highly impactful research efforts.
